# Biomineralization of Fucoidan-Peptide Blends and Their Potential Applications in Bone Tissue Regeneration

**DOI:** 10.3390/jfb8030041

**Published:** 2017-09-20

**Authors:** Harrison T. Pajovich, Ipsita A. Banerjee

**Affiliations:** Department of Chemistry, Fordham University, 441 E Fordham Rd, Bronx, NY 10458, USA; hpajovich@fordham.edu

**Keywords:** biomineralization, tissue regeneration, bone, composites

## Abstract

Fucoidan (Fuc), a natural polysaccharide derived from brown seaweed algae, and gelatin (Gel) were conjugated to form a template for preparation of biomimetic scaffolds for potential applications in bone tissue regeneration. To the Fuc–Gel we then incorporated the peptide sequence MTNYDEAAMAIASLN (MTN) derived from the E-F hand domain, known for its calcium binding properties. To mimic the components of the extracellular matrix of bone tissue, the Fuc–Gel–MTN assemblies were incubated in simulated body fluid (SBF) to induce biomineralization, resulting in the formation of β-tricalcium phosphate, and hydroxyapatite (HAp). The formed Fuc–Gel–MTN–beta–TCP/HAP scaffolds were found to display an average Young’s Modulus value of 0.32 GPa (*n* = 5) with an average surface roughness of 91 nm. Rheological studies show that the biomineralized scaffold exhibited higher storage and loss modulus compared to the composites formed before biomineralization. Thermal phase changes were studied through DSC and TGA analysis. XRD and EDS analyses indicated a biphasic mixture of β-tricalcium phosphate and hydroxyapatite and the composition of the scaffold. The scaffold promoted cell proliferation, differentiation and displayed actin stress fibers indicating the formation of cell-scaffold matrices in the presence of MT3C3-E1 mouse preosteoblasts. Osteogenesis and mineralization were found to increase with Fuc–Gel–MTN–beta–TCP/HAP scaffolds. Thus, we have developed a novel scaffold for possible applications in bone tissue engineering.

## 1. Introduction

Bone tissue is vital to the human body for structural support, including the facilitation of movement, the protection of viscera, and for regulation of mineral and acid–base homeostasis [1]. Injuries or diseases to bone tissue can significantly impact quality of life. There are a number of orthopedic conditions, including fractures, non-unions, infection, osteoporosis, osteonecrosis, metabolic bone diseases, and tumors, that result in bone loss [2]. Additionally, age-related bone volume loss, beginning as early as the third decade of life, is a common problem that is associated with a decline in fracture toughness [3,4]. Bone fractures are often treated with a cast to set the bone, however, surgery may be required [5]. Traumatic skeletal injuries including delayed unions, non-unions, and mal-unions may be healed with realignment and stable fixation of the bone for successful healing, however, bone grafting or transplant is at times necessary to initiate proper bone-healing [6]. Additionally, specific locations of the skeleton, such as the neck of the talus, the neck of the femur, and the carpal scaphoid are known to have a greater risk for impaired or delayed fracture-healing [7]. Overall, current reparative techniques are limited to bone grafts and implants [8]. Over 600,000 bone-grafting operations take place in the United States each year and is the first choice for repairing bone defects [9]. However, bone-grafting has limitations, such as donor site morbidity, limited graft supply, bleeding, chronic pain, infections, tissue rejection, disease transmission, and poor cosmetic appearances [10,11]. Tissue Engineering (TE) is a multidisciplinary field focused at restoring and regenerating injured or deficient tissue [12]. It is a promising alternative that can bypass donor tissue shortages [13] and address the limitations with current bone healing options [14]. TE was developed for a variety of organs including skin, liver, kidney, ear, neurons, bone and cornea where a patient’s own cells are harvested and cultured into a three-dimensional scaffold that is designed to mimic the extracellular matrix of the native tissue [15,16]. The strength of bone tissue, its hardness, and fracture resistance is due to the components of the bone matrix including hydroxyapatite, collagen type I, and non-collagenous proteins [17]. Accordingly, it is necessary that a designed scaffold provides structural support in a time of injury whilst also guiding and encouraging tissue growth and remodeling [18].

The exploration of biomaterials that will best promote cell growth, proliferation, and ultimately tissue regeneration is on-going and continuously challenging. Thus far, several diverse materials were proposed and developed for tissue regeneration applications such as natural and synthetic polymers, dendrimers, carbon nanotubes (CNTs), graphene oxide (GO), and peptide-based materials [19,20,21,22,23,24]. Silicate [25], phosphate [26], and borate-based [27] bioactive glasses were also utilized for bone TE. Additionally, decalcified and demineralized bone matrices with bone morphogenic proteins saw success in forming new bone in clinical applications [28,29].

In this work, we developed a new seaweed-based biocomposite for potential applications as a scaffold for bone tissue regeneration. The composite was prepared using fucoidan (Fuc), a bioactive highly branched polysaccharide extracted from marine algae [30]. It is a water-soluble heterogeneous sulfated polysaccharide that contains D-galactose, D-glucose, D-mannose, D-xylose, uronic acids and D-fucose [31,32] and has shown to have anti-inflammatory, antiviral, and anticoagulant properties as well as the ability to induce apoptosis in cancer cells in addition to arresting cell-cycle progression [33]. Additionally, it was reported that fucoidan, when combined with biopolymers such as chitosan and alginate, can form scaffolds that are highly cytocompatible and can be utilized for bone tissue regeneration [34]. Furthermore, fucoidan-tricalcium phosate-chitosan scaffolds have shown to induce differentiation of human bone marrow stromal cells [35]. Thus, we hypothesized that combining fucoidan with gelatin (Gel), a hydrolysis product of collagen [36], would result in a new scaffold template with desirable properties required for bone TE. Gelatin derived from collagen is abundantly available from sources such as mammalian skin tissue, bone, or fish skin, scales and fins, sea-urchin, and bird feet [37,38,39] and was reported to increase cell adhesion, spreading, proliferation, migration, and thus was used to enhance cell and scaffold material interactions [40]. Due to these qualities, gelatin was commonly used as a biomaterial for both soft and hard tissue engineering [41]. Moreover, gelatin is biocompatible, biodegradable, non-immunogenic, and capable of modification [42,43]. Gelatin hydrogels are formed through physical cross-linkings in water above a certain concentration and below 30–35 °C [44]. As gelatin cross-links, it aggregates and undergoes a conformational change from random coil to a triple helix [45,46,47,48].

To the Fuc–Gel composites, we next incorporated the peptide sequence MTNYDEAAMAIASLN (MTN). This sequence belongs to the EF-hand motif found in proteins, well known for their calcium binding ability [49]. This particular 15-mer sequence, was found to have exceptional ability for binding to calcium ions [50] and it was found that the amino acids T, Y and N in particular played a key role in binding to calcium, and boost the coordination ability of the peptide to Ca^+2^ by providing additional coordination sites. Thus, by incorporating a Ca^+2^ binding peptide to Fuc–Gel, the groundwork for the biomineralization of the inorganic component of bone (calcium phosphate derivative/hydroxyapatite) is accomplished by forming the Fuc–Gel–MTN composite. Biomineralization of matrices is an important milestone in the quest for organic/inorganic hybrid matrices for the regeneration of mineralized tissue [51]. In previous work, it was shown that peptide primary, secondary, and tertiary structure, as well as peptide stability, determines the mineralization influence of GEPIs [52]. Naturally occurring biopolymers such as chitin and collagen were reported to bind calcium and silica leading to biomineralization [53]. Additionally, a protein purified from *Mytilus edulis* was found to preferentially bind calcium and is responsible for the mineralization of calcium carbonate and, ultimately, the formation of the bivalve shell [54]. Furthermore, a peptide sequence derived from the salivary protein statherin showed to have a high affinity for calcium and is responsible for the nucleation of HAp [55]. More recently, genetically engineered proteins for inorganics (GEPIs) were designed through bacterial cell surface and phage display methodology [56]. In other work, it was shown that the peptide sequence EDPHNEVDGDK, derived from dentin sialophosphoprotein, strongly binds HAp nanocrystals to scaffold assemblies for bone tissue engineering [57]. Moreover, work completed by Gentile and co-workers indicated that the heparin binding domain sequences KRSR and FHRRIKA have demonstrated to adhere to cells and induce bone mineralization [58]. Gentile and co-workers also demonstrated that when the peptide sequence FHRRIKA is grafted in a nanolayer onto multilayered electrospun meshes mineralization of HAp is significantly promoted [59]. Nonoyama and co-workers showed that the peptides (LE)_8_ and (VEVSVKVS)_2_ were allowed to self-assemble forming hydrogels in the presence of calcium ions leading to HAp formation in neutral and basic pH respectively [60]. Pawelec and co-workers showed that a collagen type I based recombinant peptide (RCP) was used to create scaffolds that induced osteoblast mineralization [61].

To initiate biomineralization, the formed Fuc–Gel–MTN composite was incubated in simulated body fluid (SBF) for four weeks and the formation of calcium phosphate/apatite crystals was examined at different time points. Many reports have highlighted the use of SBF as a source for biomineralization of apatite due to its similarity with the ionic components of blood plasma [62,63,64] wherein the negatively charged phosphate groups can efficiently bind to calcium ion bound scaffolds.

Thus, we developed a new bioorganic scaffold for bone TE wherein fucoidan–gelatin assemblies were conjugated to MTN, a calcium-binding protein, forming composites capable of biphasic β-tricalcium phosphate-apatite biomineralization. Our results indicate that the formed scaffolds are mechanically and thermally stable and promoted cell proliferation and adhesion of preosteoblast cells. Additionally, alkaline phosphatase activity was enhanced, indicating cellular differentiation was promoted. Furthermore, calcium-deposits were increased in the presence of the formed scaffold as indicated by alizarin assay. Thus, we developed a highly cytocompabtible multi-layered scaffold with desirable properties for potential applications in bone tissue engineering.

## 2. Results and Discussion

### 2.1. Formation of Scaffold

**Figure 1:**
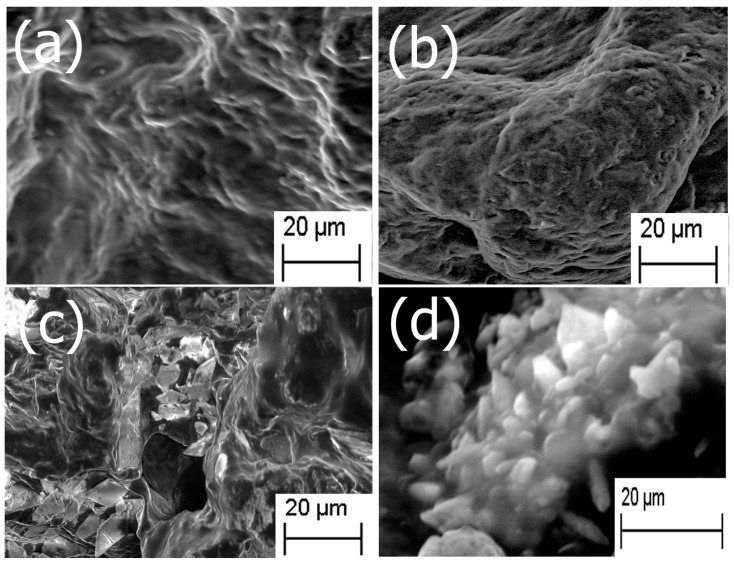
SEM images of (**a**) fucoidan-gelatin (Fuc–Gel); (**b**) Fuc–Gel–MTNYDEAAMAIASLN (MTN); (**c**) Fuc–Gel–MTN biomineralized Beta–TCP/nano HaP after 2 weeks of growth; (**d**) Fuc–Gel–MTN-biomineralized nano HaP after 4 weeks of growth in simulated body fluid (SBF).

The morphology and structural attributes of the scaffold was examined using SEM (Figure 1). Fucoidan–gelatin (Figure 1a) showed relatively gelatinous and fibrous surface. The Fuc–Gel composite is formed by covalently binding fucoidan with gelatin using NHS–EDAC coupling method. In previous work, aminated gelatin–fucoidan composites were prepared by TNBS method, which were found to form gelatinous microspheres [65] and displayed mucoadhesive properties. After attachment of the MTN peptide, we did not observe a significant change, though the surface appeared relatively rougher and small fibrous assemblies appeared to be sporadically incorporated into the matrix, confirming the formation of fucoidan–gelatin–MTN (Fuc–Gel–MTN) peptide composite (Figure 1b). The incorporation of the peptide was aided by covalent binding as well as H-bonding interactions between the -C=O and -N-H groups of gelatin and MTN. Upon biomineralization and formation of calcium phosphate, distinct changes were observed. After a two-week period of incubation with SBF, we observed the formation of plate-like crystals scattered on the surfaces of the Fuc–Gel–MTN composite (Figure 1c). After four weeks of growth, larger crystals in the dimensions of 200–500 nm were observed throughout the matrices due to biomineralization of beta-tricalcium phosphate and HAp (Figure 1d). These results indicated that Fuc–Gel–MTN was capable of inducing the growth of beta-tricalcium phosphate/hydroxyapatite crystals overtime in the presence of simulated body fluid (SBF). Previous studies showed that minerals are likely to be incorporated into gelatin sponge surfaces in the presence of SBF, thus allowing for the formation of a bone-mimetic surface, capable of functioning as cell-scaffold matrices [66,67,68]. In a separate study, poly-l-lactic acid (PLLA)/sugar scaffolds successfully formed apatite nanocrystals when incubated with SBF [69] indicating that the polymeric fibers can induce bioactivity and mineralization of nanoscale hydroxyapatite in the presence of SBF. Mineralization of collagen matrices in the presence of SBF was also reported [70]. In the case of Fuc–Gel–MTN, the MTN peptide plays a vital role of binding to calcium ions present in the simulated body fluid, allowing for the formation of a calcium-rich surface of Fuc–Gel–MTN. The MTN peptide has an isolelectric point of 3.3, making it a negatively charged peptide, and thus can bind to Ca^+2^ ions effectively. The Ca^+2^ bound Fuc–Gel–MTN then binds with the negatively charged phosphate ions present in the SBF, thus leading to the formation of calcium phosphate [71]. Because nucleation begins in aqueous solution, adsorption of water on the surface of Fuc–Gel–MTN also aids in the formation and the growth of β-tricalcium phosphate/nanoscale hydroxyapatite. Mineralization is also supported by the high ionic content of simulated body fluid—which is rich in ions such as Na^+^, K^+^, Mg^+2^, Ca^+2^, Cl^−^, HCO_3_^−^, HPO_4_^−2^ and SO_4_^2−^. It was reported that the nucleation and growth of apatite is dependent upon both the ionic concentration of SBF and surface morphology of the template scaffold [72]. In previous work, it was shown that blends of anionic sulfated glycans such as fucoidan with polymers such as polyhydroxybutyrate or polyhydroxybutarate-*co*-valerate exhibited biomineralization activity due to the negatively charged sulfate groups in fucoidan which also can efficiently bind to calcium ions [73]. Therefore, in addition to MTN peptide, calcium ion-binding is also aided by Fuc–Gel component, thereby promoting β–TCP and apatite formation. 

### 2.2. FTIR Spectroscopy

**Figure 2:**
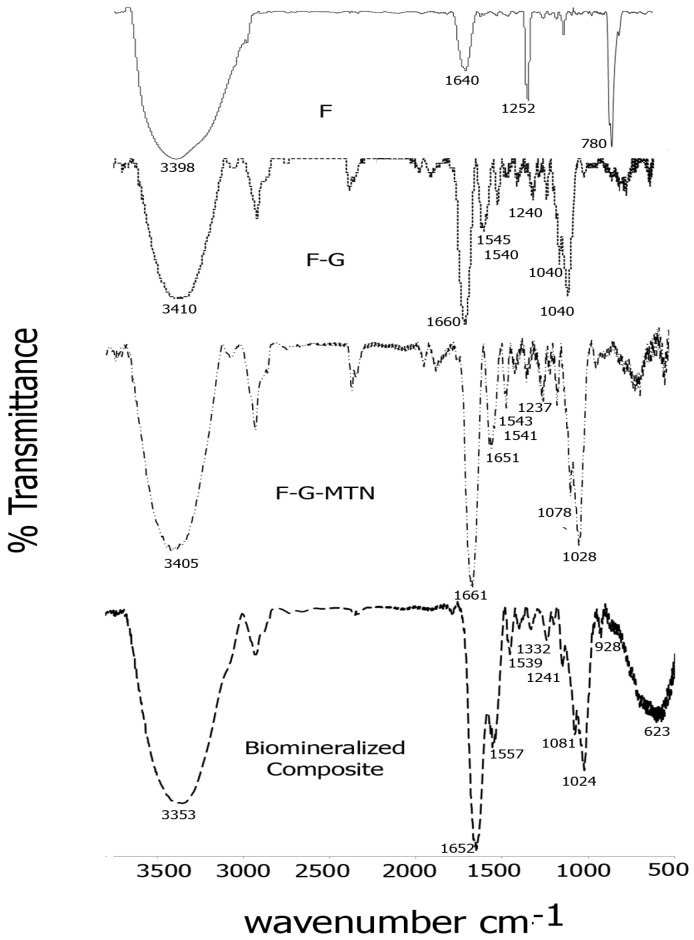
Comparison of FTIR spectra of each layer of the scaffold in the range of 500 and 3700 cm^−1^ (F = fucoidan; G = Gelatin).

The formation of Fucoidan–gelatin composites was confirmed by FTIR spectroscopy. Figure 2 shows a comparison of spectra of each of the composite layers. As seen in the figure, for the Fuc–Gel composite, we observed peaks at 3410 cm^−1^ due to -OH stretching, 2920 cm^−1^ due to -C-H stretching, strong carbonyl peaks at 1660 and at 1540 cm^−1^ due to amide I and amide II peaks respectively with a shoulder at 1545 cm^−1^, due to the presence of gelatin, a peak at 1460 cm^−1^ due to C-H bending, 1342 cm^−1^ and at 1240 cm^−1^ due to C-O stretching vibrations and a sharp peak at 1030 cm^−1^ with a shoulder at 1050 cm^−1^ due to C-H bending vibrations in sugars and C2-OH, C3-OH and C-4-OH vibrations indicating the presence of glucose residues as well as fucopyranose residues due to the presence of fucoidan [74,75,76,77]. In comparison, fucoidan alone displayed major peaks at 3398, 1640, 1252 and at 780 cm^−1^ due to -OH stretching, -C=O stretching; C-O stretching and -OH deformation respectively. A relatively short peak was found at 1039 cm^−1^ due to C-H bending vibrations in sugars [78]. These peaks were shifted considerably in comparison with Fuc–Gel composite, thus confirming its formation. Incorporation of the MTN peptide led to further changes in the FTIR peaks. The -OH peak was shifted to 3405 cm^−1^ while the amide I region showed split peaks at 1661 and at 1651 cm^−1^ and the amide II region appeared at 1541 cm^−1^ with a shoulder at 1543 cm^−1^. Peaks were observed at 1456 cm^−1^ (due to C-H bending), 1337 and at 1237 cm^−1^ due to C-O stretching. The C-H stretching vibrations due to sugars was shifted to 1078 and 1028 cm^−1^. These shifts confirm the assimilation of the MTN peptide with the Fuc–Gel composite. Upon biomineralization and formation of beta-tricalcium phosphate/HAp, the broad -OH peak was shifted to 3353 cm^−1^, while amide I and amide II peaks were observed at 1652 and at 1557 cm^−1^ with a shoulder at 1539 cm^−1^ respectively. The C-H bending peak was shifted to 1455 cm^−1^, while the C-O stretching vibration bands were shifted to 1332 and 1241 cm^−1^ respectively. The C-H bending vibrations due to sugar moieties were shifted to 1081 and 1024 cm^−1^ respectively. Peaks were observed at 928 cm^−1^ with shoulder at 918 cm^−1^ which are indicative of the phosphate group due to the formation of beta-tricalcium phosphate/HAp [79]. A broad peak is observed at 623 cm^−1^ due to bending mode of PO_4_^−3^ [80,81]. These results confirm that biomineralization occurred resulting in the formation of beta-tricalcium phosphate/HAp. 

### 2.3. XRD and EDS Analysis 

**Figure 3:**
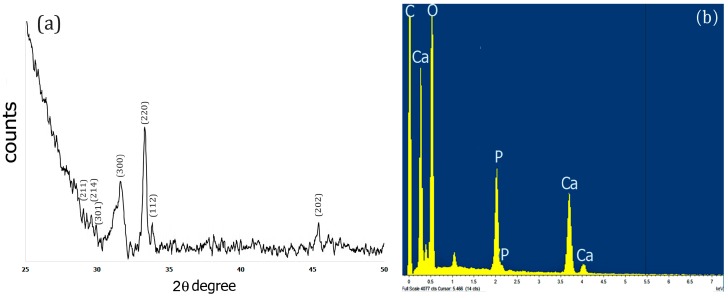
(**a**) XRD pattern of the dried Fuc–Gel–MTN-biomineralized Beta–TCP/nano HAp after 4 weeks of growth; (**b**) EDS spectrum of dried Fuc–Gel–MTN-biomineralized β–TCP/nano HAp after 4 weeks of growth.

The X-Ray diffraction pattern of the Fuc–Gel–MTN–TCP/HAp composite was obtained after drying the sample for 72 h at 250 °C. Results obtained are shown in Figure 3a. As indicated in Figure 3a, upon biomineralization, peaks were observed at 2θ = 28.9°, 29.3°, 29.9° that correspond to (211), (214) and (301) crystal phases respectively, 31.5° for (300) phase; 33.2° corresponding to (220) crystal phase; 33.9 corresponding to (112) crystal phase and at 45.4°which corresponds to (202) crystal phase. These results indicate the formation of a biphasic mixture of β-tricalcium phosphate and hydroxyapatite [82,83,84]. Similar results were obtained when fucodian hydroxyapatite composites were formed [85], though the presence of the peptide components appears to enhance crystallinity, thus confirming the formation of Fuc–Gel–MTN–βTCP/HAp composites.

To further confirm the composition of the composites, we conducted EDS analysis. As shown in Figure 3b, the presence of Ca, O and P was observed confirming the formation of Fuc–Gel–MTN–β–TCP/HAp composites. 

### 2.4. Thermal Analysis

**Figure 4:**
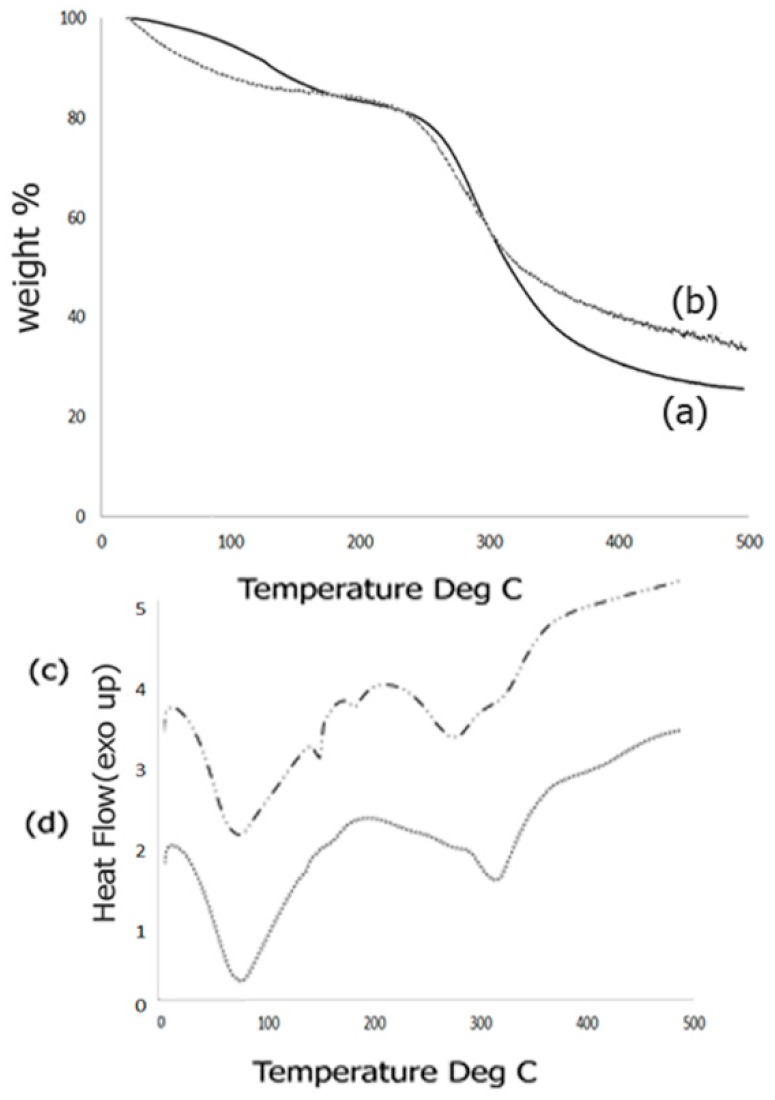
Comparison of TGA curves obtained for composite scaffold (**a**) Fuc–Gel–MTN before biomineralization and (**b**) Fuc–Gel–MTN-biomineralized β–TCP/HAp; (**c**) DSC analysis of Fuc–Gel–MTN before biomineralization and (**d**) Fuc–Gel–MTN-biomineralized β–TCP/HAp.

Thermal behavior of the scaffold composites was studied by means of TGA response curves as well as DSC analysis. The TGA response curves obtained before and after biomineralization is shown in Figure 4. The TGA curve for the Fuc–Gel–MTN before biomineralization (Figure 4a) indicated three inflection points due to the multi-component nature of the composite. The first inflection point occurred at 126.2 °C for 91% of the weight content indicating a mass loss of 9%. This is primarily due to loss of adsorbed water from the composite. The second inflection point occurred at 250.4 °C for 80% of the content indicating a mass loss of 20%. This can be attributed to gradual dehydroxylation of the fucodian and degradation of the MTN peptide and gelatin components [86]. The third inflection point was found to be 380 °C which showed a weight content of 29.5% thereby indicating a mass loss of 69.5%. This indicates that the biological components, Fuc–Gel–MTN are mostly degraded by 380 °C and residual composite Fuc–Gel–MTN (23%) remains. Similar results were observed in the case of collagen–fucodian composites [87]. Upon biomineralization, few changes were observed in the TGA curve (Figure 4b). Primarily, two inflections were observed showing a mass loss of 22% at 273 °C and 68% at 495 °C indicating that formation of beta-tricalcium phosphate and HAp leading to lesser mass loss at the higher temperature and increasing the stability of the composite [88]. Initially (below 100 °C) the mass loss was likely due to loss of water molecules due to dehydration of β–TCP/HAp. Similar results were seen in the case of hydroxyapatite containing gelatin-chitosan composites [89,90] where in upon incorporation of HAp, the gelatin-chitosan composite showed an increase in thermal stability.

We further investigated the thermal phase changes of the scaffold before and after biomineralization using DSC analysis. Phase transition behaviors of the formed scaffold from −5 to 500 °C was studied. Results obtained for samples before biomineralization are shown in Figure 4c. 

Before growth of β–TCP/HAp the Fuc–Gel–MTN composite showed a broad endothermic peak at 75.4 °C due to loss of free water, followed by short endothermic peaks at 152 and at 182.6 °C due to loss of bound water. Broad endothermic peaks are also observed at 281 and at 323 °C due to degradation and breakage of the glycosidic bonds as well as H-bonding interactions between gelatin, fucoidan and MTN. These results match with the mass losses observed during TGA analysis. Similar results were observed during pyrolysis of sulfated seaweed polysaccharides [91,92]. After biomineralization (Figure 4d), an endothermic peak is observed at 76.5 °C due to water loss, followed by another endothermic peak at 317 °C indicating that phase changes were altered upon growth of β–TCP/HAp due to changes in interactions and reorganization of H-bonds. The absence of endothermic peaks at 152, 182.6 and at 281 °C indicate that upon biomineralization the composite has altered phase changes and undergoes relatively less re-organization, indicating relatively higher stability. 

### 2.5. Mechanical and Surface Properties

**Figure 5:**
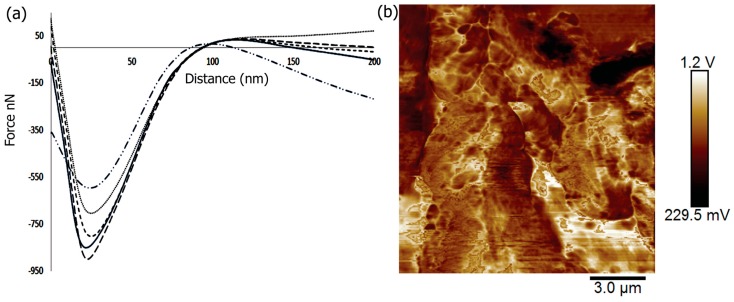
(**a**) Force distance curves of Fuc–Gel–MTN-biomineralized β–TCP/nano HAp obtained at 5 different points on the scaffold using peak force microscopy; (**b**) AFM phase image of Fuc–Gel–MTN-biomineralized β–TCP/HAp.

In order to tailor the scaffolds for bone tissue engineering, the materials must provide strength and withstand large forces. In previous work, it was shown that the mechanical properties of scaffolds could affect the induction of differentiation of cells with the stiffest scaffolds being osteogenic [93]. To investigate the mechanical properties of the Fuc–Gel–MTN-biomineralized beta–tricalcium phosphate/HAp scaffold, we conducted peak force microscopy using AFM. The results obtained are shown in Figure 5. The scaffolds were first imaged in peak force tapping mode and the cantilever was positioned at different points on the scaffold before examining mechanical properties. On average, at least three force-displacement curves at various positions on the scaffold were obtained. Force versus displacement obtained at three different regions on the scaffold are indicated in Figure 5a. We determined the Young’s Modulus (YM) by fitting the data to the Hertzian model. The average Young’s Modulus was found to be 0.302 ± 4 GPa. This value is higher than that reported for gelatin and gelatin-PCL nanofibers [94] due to the formation of a multilayered scaffold containing β–TCP/HAp. However, the YM value obtained is lower than that obtained for poly (lactic-*co*-glycolic acid) (PLGA) loaded nanodiamond phospholipid matrices, which displayed a value of 5.7 GPa [95]. In general, it is known that the elastic moduli obtained using AFM-based nanoindentation studies are lower than those obtained by macroscopic torsional tests due to differences in behavior of materials at the macroscopic and microscopic levels, where different interactions come into play in addition to differences in structural deformation [96]. The literature values reported for YM of trabecular bone varies between 0.7 to 20 GPa, while that of cortical bone was reported to be in the range of 5–27 GPa depending on the structural aspects and technical methods utilized [97]. Thus, the fucoidan–Gel–MTN–TCP/HAp may be more suitable for trabecular bone tissue regeneration. 

In addition to nanoindentation studies, we also measured the surface roughness of the biomineralized composite. It was shown that increased roughness aids in forming an interface between the scaffold and native bone tissue [98]. Additionally, surface roughness allows for the adhesion as well as proliferation of osteoblasts and increased collagen synthesis [99,100]. Average surface roughness (Ra), root-mean-square roughness (Rq) and the maximum roughness (Rmax) for the Fuc–Gel–MTN-biomineralized HAp scaffold were determined. The Ra value was found to be 91 ± 11 nm and Rq was found to be 106 ± 12 nm, while Rmax was found to be 443 ± 18 nm. Figure 5b shows the AFM phase image further confirming the multilayered, porous and three-dimensional qualities of the scaffold.

### 2.6. Rheology

**Figure 6:**
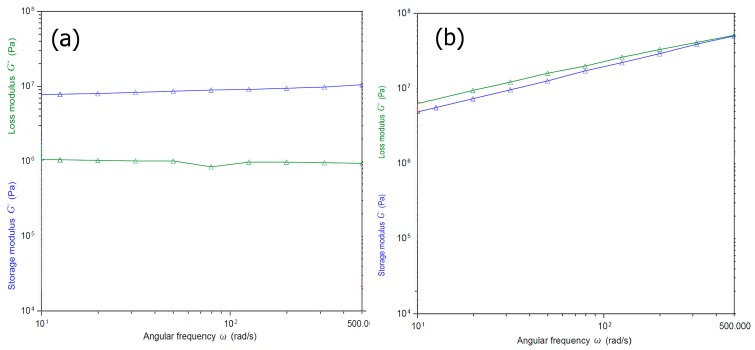
Angular frequency (ω) dependence of storage (G′) and loss modulus (G″) (**a**) before and (**b**) after biomineralization of the biocomposite.

To examine the viscoelastic properties of the Fuc–Gel–MTN–beta–TCP/HAp, we carried out rheology studies by conducting oscillatory shear measurements. The samples were subjected to sinusodial strain at an angular frequency range from 10 to 500. In general, it was reported that incorporation of fucoidan results in an increase in viscoelasticity, particularly in the presence of salts such as sodium chloride or calcium chloride [101]. Each study was carried out thrice. As shown in Figure 6, we measured the storage and loss moduli G’ and G”. Before biomineralization, (Figure 6a) in the case of the Fuc–Gel–MTN the storage modulus was found to be approximately one order of magnitude higher than the loss modulus and very little change was observed due to frequency changes; which indicates the formation of a gel structure [102]. The loss modulus also was found to be relatively independent of frequency, except a slight dip in modulus at an angular frequency of 100. The high values of the modulus indicates that the elasticity is primarily due to the inherent stiffness and cross-linking of the gel. Figure 6b shows the storage and loss moduli for the biomineralized composite. As seen in the figure, the storage and loss moduli were parallel, and both showed a slight decrease in values throughout the frequency range studied. Furthermore, the magnitude of difference between storage and loss moduli was fairly small at lower frequencies with storage modulus being 1/10th of an order lower than the loss modulus at lower frequencies indicating higher visco-elasticity. However, after biomineralization, the composite has higher storage and loss higher modulus overall due to formation of β–TCP/HAp within the scaffold matrices, indicating higher strength and ability to store deformation energy due to less mobility within the gel as a result of beta–TCP/HAp being inter-dispersed within the composite.

### 2.7. Cell Studies

#### 2.7.1. Cell Viability and Morphology Studies

**Figure 7:**
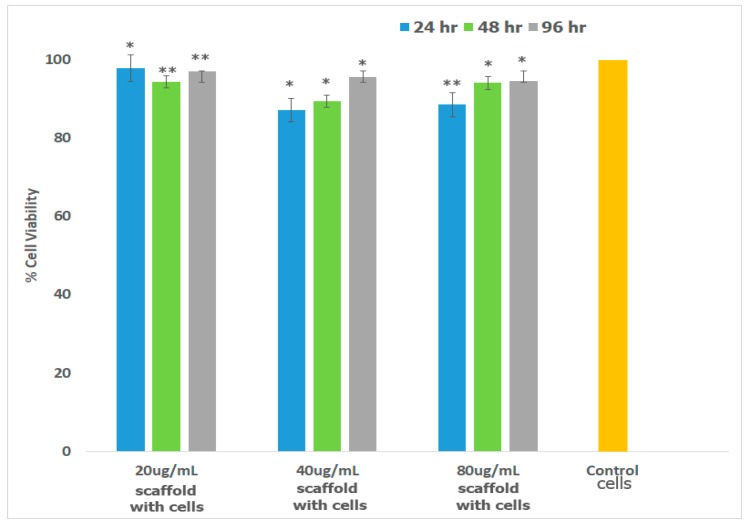
Percent Cell Viability of MC3T3-E1 cells in the presence of varying amount of scaffolds was carried out over a period of 96 h. Measurements were made after 24, 48 and 96 h using MTT assay. Each bar in the figure represents the mean of three independent studies with standard deviation (SD). Significant difference was analyzed by comparing the viability control with those of cells in the presence of scaffolds. * indicates *p* < 0.05; ** represents *p* < 0.01. The *p* values were determined by student’s *t*-test.

Preosteoblasts, or osteoprogenitor cells, develop from the bone marrow and are responsible for the repair and regeneration of bone tissue [103]. Osteoblasts possess the enzyme Alkaline Phosphatase which catalyzes mineralization and bone formation making osteogenesis possible [104]. To confirm that the scaffold is well-suited for bone TE, we investigated cell viability in the presence of mouse preosteoblasts (MT3C3-E1) cell lines. The results obtained are shown in Figure 7. As illustrated in the figure, cells continued to proliferate over 96 h similar to the control. Relatively slower growth was observed for cells incubated with 40 μg/mL and 80 μg/mL scaffold at 24 h, but growth was comparable at 48 and 96 h. Overall growth in all cases after 96 h was >92%. 

**Figure 8:**
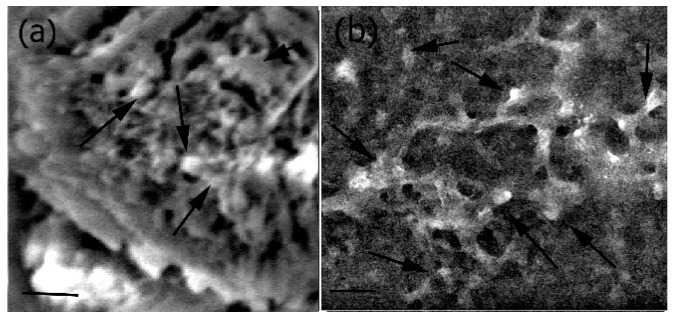
Interactions of MC3T3-E1 cells with Fuc–Gel–MTN-biomineralized β–TCP/HAp matrix showing formation of cell-scaffold matrices (**a**) in the presence of 20 μg/mL Fuc–Gel-MTN-biomineralized HAp; (**b**) in the presence of 40 μg/mL Fuc–Gel–MTN-biomineralized β–TCP/HAp. Scale bar for (**a**) = 30 μm; (**b**) = 30 μm. Arrows indicate cellular attachments.

To further evaluate the interactions of the cells with the Fuc–Gel–MTN–β–TCP–HAp scaffold, cells were imaged after incubation with different quantities of scaffolds for 48 h as shown in Figure 8. As seen in the figure, in both cases (presence of 20 μg/mL scaffold (Figure 8a) and 40 μg/mL scaffold (Figure 8b)), three dimensional multi-layered cell-scaffold matrices formed after cells were seeded with the scaffolds. In general, cells were found distributed and well spread out throughout the matrices and were attached to the scaffolds, though a denser layer of cells was observed on the upper layers of the scaffolds. 

#### 2.7.2. Cytoskeletal Staining (Phalloidin Assay)

**Figure 9:**
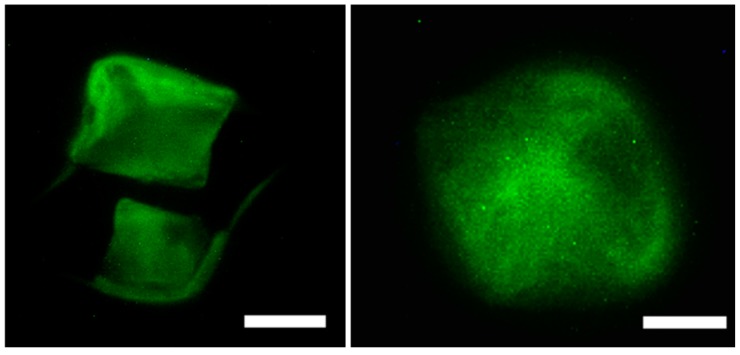
Organization of actin stress fibers of MC3T3-E1 cells upon interacting with the scaffolds indicating that the scaffold strongly adhered to preosteoblasts in the presence of 20 μg/mL of scaffold (**left**) and in the presence of 40 μg/mL scaffold (**right**) Scale bar = 50 μm (**left**); 30 μm (**right**).

To examine the binding interactions and cytoskeletal effect of the scaffolds on the actin cytoskeleton of the cells, we performed cytoskeletal staining assay using cruz-fluor conjugated phalloidin. Focal adhesion points, polymerization of actin and formation of actin stress fibers, are reliant on the immediate cellular environment and interactions of cells with scaffolds [105] and play a critical role in maintaining cell motility and overall mechanics of cells. Thus, important information regarding binding interactions involved in cell-scaffold matrix formation can be obtained using this assay. Figure 9 demonstrates the actin cytoskeletal arrangement of MC3T3-E1 cells after incubation with varying amounts of scaffolds for 24 h. The results reveal that the actin fibers appeared more toward the periphery of cells at the lower scaffold concentration (20 μg/mL) thus allowing the cells to adhere to the scaffolds. More actin stress fibers, at the periphery as well as at the center of the cells which are connected through focal adhesion points were visible in the presence of the 40 μg/mL of the scaffold most likely due to interactions with higher quantities of the scaffold. This in turn allows cells to be well spread throughout the scaffold. These results further confirm that the Fuc–Gel–MTN–beta–TCP/HAp scaffolds are interacting with preosteoblasts causing the rearrangement of actin fibers [106] and successfully adhered to the scaffolds.

#### 2.7.3. Alkaline Phosphatase Assay

**Figure 10:**
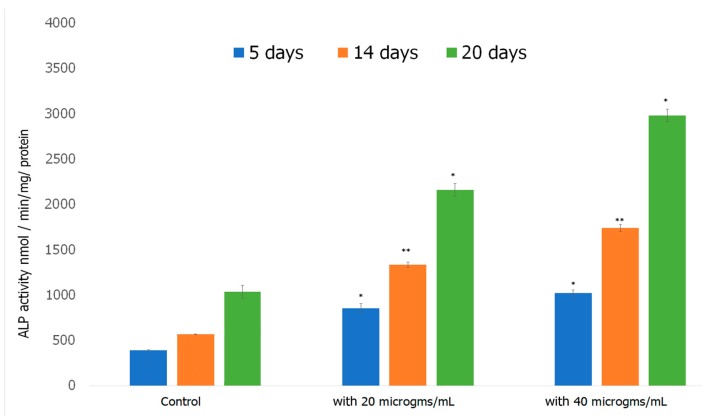
Alkaline phosphatase (ALP) activity was measured in MC3T3-E1 cells before and after incubation with the scaffolds over a period of 21 days. Values are standardized by the total amount of protein in the sample. Results are indicated as standard deviation of three experiments performed in. * *p* < 0.05, ** *p* < 0.01, significantly different from control cells.

Alkaline Phosphatase Assay was performed to investigate the ability of preosteoblasts to differentiate in the presence of the formed scaffold. Alkaline Phosphatase (ALP) is an early biomarker for osteoblast differentiation [107]. ALP catalyzes the hydrolysis of pyrophosphate, initiating the mineralization of HAp [108]. ALP activity increases as cells advance from proliferation stage to ECM deposition stage thus plays a vital role in the formation of HAp, the major mineral in bone tissue responsible for the stiffness and strength of bone [109]. The results obtained over a 20 day period of incubating cells with the scaffold are shown in Figure 10. ALP activity increased considerably in preosteoblasts incubated with both 20 and 40 μg/mL of the formed Fuc–Gel–MTN-biomineralized β–TCP-HAp scaffolds compared to preosteoblasts alone. At 5 days, the 20 and 40 μg/mL treated cells exhibited significantly higher ALP activity compared to the control. For the cells treated with 20 μg/mL scaffold, ALP activity did not increase significantly between 5 and 14 days, while the 40 μg/mL treated cells, ALP activity doubled between 5 and 14 days. At the 20th day time point, samples incubated with the formed scaffolds had more than doubled their ALP activity compared to the 14 day activity. In all cases, higher activity was observed in the presence of the scaffold compared to cells alone. The increased ALP activity indicates cell differentiation had occurred, thus the formed scaffolds possess a strong ability to differentiate preosteoblasts into osteogenic lineage, which is essential for bone tissue formation. 

#### 2.7.4. Alizarin Assay

**Figure 11:**
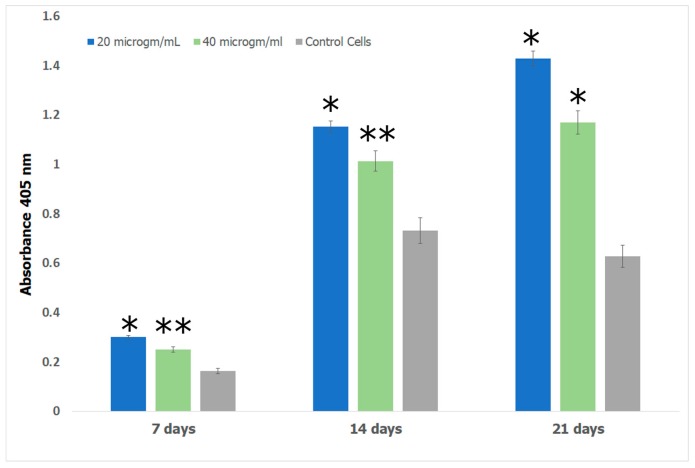
Alizarin S quantification using the assay carried out for MC3T3-E1 cells showing induction of osteogenesis in the presence and absence of different quantities of Fuc–Gel–MTN–beta–TCP/HAP scaffolds. Data are expressed as the mean (*n* = 3) with error bars indicating standard deviations. * *p* < 0.05; ** *p* < 0.01, significantly different from control cells.

**Figure 12:**
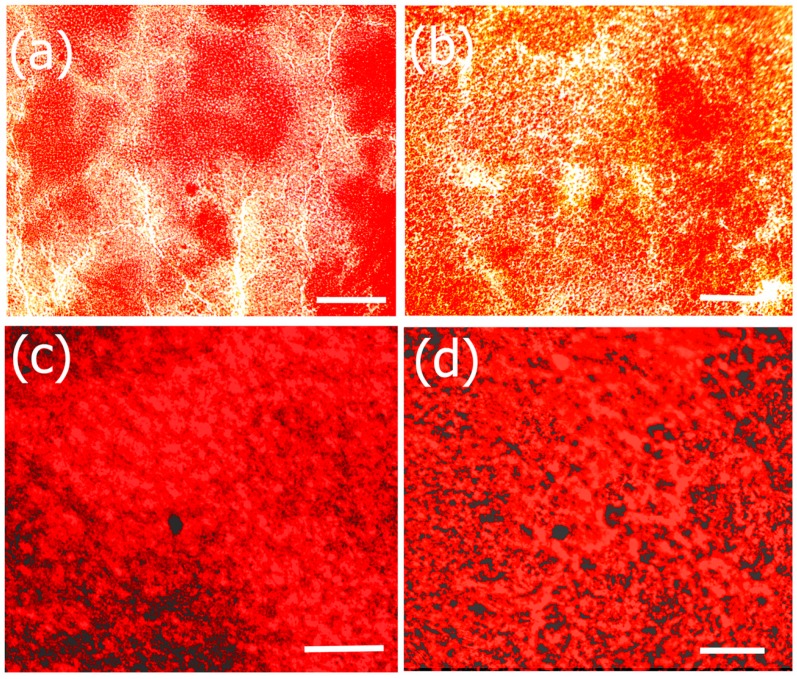
Alizarin S staining (**a**,**c**) indicating staining and formation of calcium deposits after 7 and 21 days in the presence of 20 μg/mL of scaffold and preosteoblasts; (**b**,**d**) indicate staining and formation of calcium deposits after 7 and 21 days in the presence of 20 μg/mL of scaffold and preosteoblasts. Scale bar = 100 μm.

Alizarin red S (ARS) assay was performed to investigate the formation of calcium deposits by preosteoblasts. Bone growth occurs in the lacunae of bone tissue through calcification [110]. In previous work, it has been shown that the lacunae in growing bone attracts calcium cations which behave as nucleating agents for the formation of HAp [111]. We therefore explored the potential of the Fuc–Gel–MTN–βTCP–HAp for formation of calcium deposits and osteogenesis using alizarin assay. The obtained results are shown in Figure 11. The results show that cells incubated with the Fuc–Gel–MTN–beta–TCP/HAp scaffolds formed more calcium deposits than preosteoblasts alone at 7, 14, and 21 days. Overall, slightly higher calcium deposits were observed in the presence of 20 μg/mL of scaffold. This may be due to higher matrix secretion and faster calcification in those cases. Regardless, in both cases deposition calcium increased over time, indicating osteogenic differentiation. Figure 12 shows the red staining of the samples indicating the formation of calcium deposits by the preosteoblasts at 7 and 21 days in the presence of 20 and 40 μg/mL scaffold. 

## 3. Materials and Methods

### 3.1. Materials

*N*-Hydroxy Succinimide (NHS) and 1-ethyl-3-(3-dimethylaminopropyl) carbodiimide (EDAC) were purchased from Sigma-Aldrich (St. Louis, MO, USA). Porcine Skin Gelatin Type A (Gel Strength 300) and Fucoidan from Fucus Vesiculosus were purchased from Sigma Aldrich (St. Louis, MO, USA). The calcium-binding peptide (MTN) MTNYDEAAMAIASLN was custom ordered from GenScript (Piscataway, NJ, USA). Mouse Preosteoblasts (MC3T3-E1, lot 63465740, (Manassas, VA, USA) were purchased from ATCC (Manassas, VA, USA) and cell culture media was purchased from ATCC (Manassas, VA, USA) or Thermofisher Scientific (Springfield, NJ, USA). Osteoblast Mineralization medium (C-27020) and Osteoblast growth media supplement mix (C-39615) as well as the basal medium (C-27010) were purchased from PromoCell (Heidelberg, Germany). Phalloidan Cruz Fluor 488 Conjugate was purchased from Santa Cruz Biotechnology (Dallas, TX, USA). MTT Assay kit was purchased from Cayman Chemical Company (Ann Arbor, MI, USA). Alkaline Phosphatase Assay Kit was purchased and Aliziran Red S Solution pH 4.2 were purchased from Thermofisher Scientific (Springfield, NJ, USA).

### 3.2. Preparation of Scaffold

Gelatin (10 mM) and Fucoidan (5 mM) were allowed to react by coupling the carboxylate groups of fucoidan with the amine groups of gelatin in the presence of NHS (0.5 mM) and EDAC (0.5 mM) in distilled water under aqueous conditions at 4 °C for 3 h. Fuc–Gel assemblies were then centrifuged and allowed to react in the presence of NHS (0.5 mM) and EDAC (0.5 mM) with the MTN peptide and the mixture was stirred at 4 °C for 24 h. The Fuc–Gel–MTN composites were then incubated in Simulated Body Fluid (SBF) at 4 °C for a period of three weeks and the morphologies of the obtained scaffolds were examined every week. SBF was prepared according to previously established methods [112]. The SBF was prepared as follows: Briefly, distilled water (750 mL) was heated to a constant temperature of 36.5 °C. A magnetic stirrer was then used to dissolve the reagents in the following order: 7.996 g of NaCl, 0.350 g NaHCO_3_, 0.224 g KCL, 0.228 g K_2_HPO_4_ 3H_2_O, 0.305 g MgCl_2_·6H_2_O, 40 mL HCl (1 M), 0.278 g CaCl_2_, 0.071 g Na_2_SO_4_, 6.057 g (CH_2_OH)_3_CNH_2_. To correct the pH of the solution to pH 7.4, 1 M HCl solution was added. The total volume of the solution was then brought to 1 L using distilled water. The solution was cooled at room temperature and stored at 4 °C for 24 h prior to use.

### 3.3. Characterization

#### 3.3.1. Scanning Electron Microscopy (SEM)

Analyses were carried out using a Zeiss EVO MA10 model. Samples were dried on to carbon double stick conducting tapes and were examined at a range of 5 to 10 kV at varying magnifications. In general the instrument was operated in EP mode. 

#### 3.3.2. Energy Dispersive X-ray Spectroscopy (EDS)

The chemical composition of the biomineralized Fuc–Gel–MTN–β–TCP–HAp was evaluated by EDS analysis using Silicon Drift detector extreme (SDD Extreme, Oxford Instruments, Abingdon, UK) from Oxford instruments, connected to the Zeiss EVO MA10 SEM (Carl Zeiss Inc., Thornwood, NY, USA) under vacuum. Samples were fixed on stubs with carbon tape. Several areas on the SEM image were selected after acquisition of the images. In general for EDS analysis, all SEM images were acquired at 15 kV in HV mode. Quantitative analysis was carried out using Aztec energy analysis and EDS analysis software (Oxford Instruments, Abingdon, UK).

#### 3.3.3. Fourier Transform Infrared (FTIR) Spectroscopy

Fourier transform infrared spectroscopy was conducted after incorporation of each layer using a Thermo Scientific, Nicolet IS50 FTIR (Thermo Scientific, Waltham, MA, USA) with OMNIC Software (Thermo Scientific, Waltham, MA, USA). KBr pellets of samples were prepared and all spectra were taken at 4 cm^−1^ resolution with 100 scans for averaging. The sample measurements were taken between 400 and 4000 cm^−1^. 

#### 3.3.4. Thermogravimetric Analysis

Thermogravimetric analysis of the scaffolds before and after biomineralization in SBF was carried out using TA Instruments Q500 TGA (TA Instruments, New Castle, DE, USA). Samples were dried in vacuum prior to analysis. In general studies were carried out under nitrogen at a heating rate of 10 °C per minute. Studies were carried out in the range of 20 to 500 °C. Each study was carried out thrice. 

#### 3.3.5. Differential Scanning Calorimetry (DSC)

To examine thermal phase changes, DSC analysis of the scaffold was carried out before and after biomineralization in SBF using TA Instruments Q200 DSC (TA Instruments, New Castle, DE, USA). Studies were carried out in the range of 0 to 500 °C under nitrogen flow at the rate of 10 °C per minute. Samples were dried under vacuum prior to analysis. In general samples in the range of 1 to 2 mg were used. Each study was carried out in triplicate. 

#### 3.3.6. X-ray Diffraction (XRD)

X-ray diffraction was carried out to investigate the crystallinity of the samples using a Bruker D8 Eco Advance (Bruker, Billerica, MA, USA) with a with a monochromatic CuKα radiation. Samples were analyzed at a range of 2θ angle between 20° to 60° with a scan rate of 1.5° min^−1^. Samples were dried and powdered and placed on sample holder prior to analysis. 

#### 3.3.7. Atomic Force Microscopy

Atomic force microscopy was performed using a Bruker MultiMode 8 HR (Bruker, Billerica, MA, USA). Imaging was carried out using peak force microscopy (PFM). Peak force microscopy in QNM mode was carried out using RTESPA-525 0.01–0.025 Ohm-cm Antimony (n) (Bruker, Billerica, MA, USA) doped Si cantilever with spring constant 200 N/m to determine nanomechanical properties and surface roughness of the scaffold. To determine Young’s Modulus, data were fitted to a Hertzian model. Samples were allowed to dry onto freshly cleaved mica sheets (Grade V-1, thickness 0.15 mm, size 15 mm × 15 mm) (Electron Microscopy Sciences, Hatfield, PA, USA) before mounting on the AFM.

#### 3.3.8. Rheology

Rheology measurements of the formed scaffold before and after biomineralization was carried out using a Discovery Hybrid HR2 Rheometer (TA instruments, New Castle, DE, USA). Measurements were carried out 25 °C on a peltier plate using an 8 mm peltier cone geometry. Samples were analyzed between angular frequencies ω of 10 and 500 rad/s. Samples were air-dried before analysis. Measurements were carried out in triplicate in air.

### 3.4. Cell Studies

Mouse preosteoblasts (MC3T3-E1 cells) were grown to confluence in Dulbecco’s Modified Eagle’s Medium (DMEM; GIBCO), supplemented with 10% fetal bovine serum (Thermofisher Scientific), 1% 10,000 I.U./mL Penicillin, 10,000 (μg/mL)100 units/mL streptomycin. Cells were grown as monolayers in a humidified atmosphere at 37 °C in a 5% CO_2_ incubator and washed and split every two days. 

#### 3.4.1. Cell Viability and Morphology Studies

To examine cell viability in the presence and absence of the Fuc–Gel–MTN–biomineralized HAp scaffolds, we plated cells at a density of 1 × 10^4^ cells/well in 96-well Falcon polystyrene tissue culture plates. After allowing the cells to spread and attach for 3 h, we added Fuc–Gel–MTN–biomineralized HAp scaffolds at varying concentrations to the wells. We tested 20, 40, and 80 μg/mL concentrations of the scaffold. The growth of the cells was monitored over a period of 24, 48, and 96 h. To determine cell viability, we performed MTT assay. The absorbance at 570 nm was monitored at each time point using a BioTek Eon microplate reader. Triplicate experiments were run in all cases. The absorbance of media alone was used at the blank and was subtracted from all samples. Percent cell viability was calculated according to the formula [(O.D of cell plus scaffolds)/(O.D of cells alone)] × 100. The standard deviations were calculated. Statistically significant differences were then determined using student’s *t*-test. 

##### SEM Imaging of Cells

Silicon chips (Ted Pella) (5 mm × 5 mm) were irradiated with UV-light and washed with ethanol and dried and coated with poly-l-lysine. They were then placed in 6 well plates. Mouse preosteoblasts at a cell density of 1 × 10^3^ cells were plated on the silica chips, followed by the addition of either 20 or 40 μg/mL of the formed scaffolds, followed by the addition of 2 mL of media. The cells and scaffolds were allowed to interact for 48 h, after which the media was removed and cells were washed with PBS. Cells were then fixed with 2% glutaraldehyde in NaHCA buffer (30 mM HEPES, 100 mM NaCl, 2 mM CaCl_2_) for 1.5 h at room temperature. The fixed cells were rinsed with PBS and then post-fixed with 1% OsO4 in PBS for 1 h at room temperature in the dark. The cells were then rinsed with distilled water and dehydrated through 5 min room temperature steps of washing with 50%, 75%, 95%, and 100% ethanol. The cell-scaffold matrices were then imaged using SEM, by directly putting the silicon chip on to the SEM stub using a carbon double stick tape. 

#### 3.4.2. Cytoskeletal Staining (Phalloidan Assay)

To further examine the effect of the scaffolds on the cytoskeleton of cells, we conducted phalloidin assay. Stock solution of Phalloidin CruzFluor conjugate staining solution (1000×) was prepared DMSO following manufacturer’s protocol. Then 1 µL of 1000× Phalloidin CruzFluor stock conjugate in DMSO was added to 1 mL of PBS with 1% BSA. Cells were fixed following the modification of previously established methods [113]. Briefly, cells with varying concentrations of scaffold were allowed to spread and grow to confluence in 6-well culture plates for 24 h on a coverslip that was coated with poly-l-lysine. After removal of media, cells were washed twice in PBS. After removal of PBS, the cells were incubated with the staining solution (phalloidin conjugated with cruz-fluor-488) and allowed to sit undisturbed for 20 min. The cells were subsequently fixed using 1 m: of 4% paraformaldehyde (PFA) solution for 20 min at room temperature and then washed thrice with PBS. The cells were then permeabilized using Triton-X (Thermo Fisher Scientific, Waltham, MA, USA) (0.1% WT) for 15 min at room temperature. The cells were then washed thrice with PBS and incubated with PBT (PBS + 0.1%WT Tween-20) for 30 min. Samples were then imaged using FITC filter (488 nm) on a Leitz Laborlux S fluorescence microscope.

#### 3.4.3. Alkaline Phosphatase Assay

To assess the differentiation ability of the MC3T3-E1 preosteoblast cells, alkaline phosphatase assay was conducted to quantify the activity of alkaline phosphatase enzyme. Mouse preosteoblasts were plated in 6-well plates at a density of 1 × 10^4^ cells per well. 3 × 3 sets of well plates were prepared to examine the alkaline phosphate activity over a period of 21 days. Cells were allowed to spread and attach for 1 h and either 20 or 40 µg/mL of the formed scaffold was added. After 4 days, the media was changed to Osteoblast growth media (PromoCell, Heidelberg, Germany) to which osteoblast growth medium supplement mix (PromoCell) was added. The osteoblast growth media plus supplement mix was changed every two days throughout the course of study. Alkaline phosphatase assay was carried out on day 5, day 14 and day 20 for each set. In general to conduct the assay, cells were washed thrice with PBS and lysed with 0.2% Triton X-100 and shaken for 20 min at room temperature. 25 µL of these samples were added to 96 well plates containing 175 μL working solution. Working solution was prepared by combining 200 μL Assay buffer, 5 μL Mg Acetate, and 2 μL PNPP (p-nitrophenyl phosphate). The plates were tapped briefly to mix and absorbance was read at 405 nm at times t = 0 and t = 4 min using a BioTek Eon microplate reader (Biotek, Winooski, VT, USA). The alkaline phosphatase activity in the supernatant was determined using instructions given in the ALP assay kit (Cayman Chemicals, Ann Arbor, MI, USA) with p-nitrophenyl phosphate as substrate. The enzyme activity was calculated according to protocols given by the manufacturer.

#### 3.4.4. Alizarin Assay

To examine the formation of calcium-deposits and study the osteogenesis inducing capability of the scaffolds for the MC3T3-E1 cells, alizarin assay was conducted. Mouse preosteoblasts were plated in 24-well plates at a density of 1 × 10^4^ cells/well with Osteoblast Mineralization medium which was pre-mixed with supplement mix (PromoCell). 3 × 3 sets of cells were plated in order to carry out tests at 7, 14 and 21 day time points. 3 sets were prepared for examining the formation of calcium deposits via imaging. In general, cells were allowed to spread and attach for 1 h and varying concentrations of scaffold were added. Either 20 or 40 µg/mL of the formed scaffold was added. Alizarin assay was conducted at each time using protocols suggested by the manufacturer. Briefly, to assess calcium deposits cells were washed thrice with PBS and subsequently fixed in 4% Paraformaldehyde for 15 min at room temperature. The cells were then washed thrice with dH_2_O. 250 μL of 40 mM ARS was added and cells were shaken at room temperature for 30 min. The stain was then removed and cells were washed five times with dH_2_O. Optical images were taken using an IN200TA-P Inverted tissue culture microscope (AmScope, Irvine, CA, USA) with USB camera. 

To quantify Alizarin S, 10% acetic acid was added to each well and incubated at room temperature for 30 min with shaking. The cells were collected using a cell scraper and the cells were transferred in 10% acetic acid to a 1.5 mL microcentrifuge tube and vortexed for 30 s. Samples were heated at 85 °C for 10 min. The centrifuge tubes were then incubated on ice for 5 min and centrifuged at 20,000× *g* for 15 min. 500 μL of supernatant of each sample was added to a new microcentrifuge tube and 10% ammonium hydroxide was added. 150 μL of each sample was then added to a 96-well plate and the absorbance was read at 405 nm using a BioTek Eon microplate reader (Biotek, Winooski, VT, USA). The amount of mineralization produced was quantified using a standard curve for ARS.

### 3.5. Statistical Analysis

All studies were carried out in triplicate. To determine significant difference, we used Student’s *t*-test method. 

## 4. Conclusions

In this work, a new multilayered fucoidan–gelatin–MTN–β–TCP–HAp scaffold was fabricated for potential applications in bone tissue engineering applications. It was found that fucoidan–gelatin–MTN bound to calcium ions and efficiently formed biphasic β–TCP–HAp in the presence of simulated body fluid. The formation of the scaffolds was confirmed by SEM and AFM analysis as well as FTIR spectroscopy. Nanomechanical properties were studied to determine the Young’s Modulus and surface roughness of the formed scaffold. The viscoelastic properties as well as thermal phase changes of the scaffold were examined before and after biomineralization. Results indicated that biomineralization of the scaffolds lead to increased stability and higher storage and loss modulus, indicating the formation of a harder, stiffer material. The scaffolds were found to adhere to preosteoblasts and formed three dimensional cell-scaffold matrices. Furthermore, the scaffolds promoted cell proliferation, differentiation and osteogenesis. Thus the biocomposite designed herein, derived entirely from naturally available sources may open new doors for preparation of scaffolds that may be utilized for bone tissue engineering applications.
